# Molecular Dynamics of Channelrhodopsin at the Early Stages of Channel Opening

**DOI:** 10.1371/journal.pone.0131094

**Published:** 2015-06-26

**Authors:** Mizuki Takemoto, Hideaki E. Kato, Michio Koyama, Jumpei Ito, Motoshi Kamiya, Shigehiko Hayashi, Andrés D. Maturana, Karl Deisseroth, Ryuichiro Ishitani, Osamu Nureki

**Affiliations:** 1 Department of Biological Sciences, Graduate School of Science, The University of Tokyo, 7-3-1 Hongo, Bunkyo-ku, Tokyo 113–0033, Japan; 2 Department of Bioengineering Sciences, Graduate School of Bioagricultural Sciences, Nagoya University, Furo-cho, Chikusa-ku, Nagoya, 464–8601, Japan; 3 Department of Chemistry, Graduate School of Science, Kyoto University, Kyoto 606–8502, Japan; 4 Department of Bioengineering and Howard Hughes Medical Institute, Stanford University, Stanford, California 94305, United States of America; Universidad Autonoma de San Luis Potosi, MEXICO

## Abstract

Channelrhodopsin (ChR) is a light-gated cation channel that responds to blue light. Since ChR can be readily expressed in specific neurons to precisely control their activities by light, it has become a powerful tool in neuroscience. Although the recently solved crystal structure of a chimeric ChR, C1C2, provided the structural basis for ChR, our understanding of the molecular mechanism of ChR still remains limited. Here we performed electrophysiological analyses and all-atom molecular dynamics (MD) simulations, to investigate the importance of the intracellular and central constrictions of the ion conducting pore observed in the crystal structure of C1C2. Our electrophysiological analysis revealed that two glutamate residues, Glu122 and Glu129, in the intracellular and central constrictions, respectively, should be deprotonated in the photocycle. The simulation results suggested that the deprotonation of Glu129 in the central constriction leads to ion leakage in the ground state, and implied that the protonation of Glu129 is important for preventing ion leakage in the ground state. Moreover, we modeled the 13-*cis* retinal bound; i.e., activated C1C2, and performed MD simulations to investigate the conformational changes in the early stage of the photocycle. Our simulations suggested that retinal photoisomerization induces the conformational change toward channel opening, including the movements of TM6, TM7 and TM2. These insights into the dynamics of the ground states and the early photocycle stages enhance our understanding of the channel function of ChR.

## Introduction

Most organisms perceive light-carried information by using rhodopsin family proteins, which are covalently bound to a retinal chromophore. During evolution, rhodopsin family proteins have acquired divergent functions, as light sensors, ion pumps, and ion channels. Channelrhodopsin (ChR) is the only channel-type rhodopsin family protein identified thus far. ChR was originally isolated from *Chlamydomonas reinhardtii*, and was characterized as a light-gated cation channel in 2002 [1]. Upon excitation by blue light, ChR permeates several monovalent and divalent cations, including H^+^, Na^+^, K^+^, and Ca^2+^. As the net inward flow of cations under physiological ionic conditions depolarizes cell membranes, ChR has become not only an attractive target of biophysical studies, but also a powerful tool for the neuroscience field (optogenetics). Since ChR was first expressed in mammalian neurons in 2005 [2,3], the number of studies using ChR has rapidly increased [4]. The integration of ChR and fiber-optic devices allows the control of neural activity with high spatial and temporal precision, even within systems such as freely moving mammals. Thus, ChR is now recognized as a powerful tool for modulating neural activity, in order to elucidate the neural circuit foundations of normal and pathological behaviors [4,5]. However, despite the rapid progress in optogenetics [4], our understanding of the mechanism by which ChR permeates cations in response to light is still limited.

Previous electrophysiological analyses of ChR revealed that its N-terminal extracellular domain (N-domain) and seven-transmembrane (TM) domain are sufficient for the channel function, while its large C-terminal intracellular domain is dispensable [1,6]. In the center of the TM domain, an all-*trans* retinal (ATR) is covalently bound to Lys296 (Lys257 in ChR2) on TM7, thus forming the protonated Schiff base, and the positive charge of the Schiff base is stabilized by two counterions, Glu162 and Asp292 (Glu123 and Asp253 in ChR2, respectively). Upon receiving blue light, the all-*trans* retinal (ATR) isomerizes to 13-*cis* retinal (13-*cis*R), which triggers a series of chemical reactions and conformational changes, called the photocycle. Electrophysiological and spectroscopic experiments revealed that the ChR photocycle comprises the early photoproducts, P_1_^500^ and P_2_^390^, the conducting state, P_3_^520^, the late intermediate state, P_4_^480^, and other intermediates in the branched side pathways [7–12].

**Fig 1:**
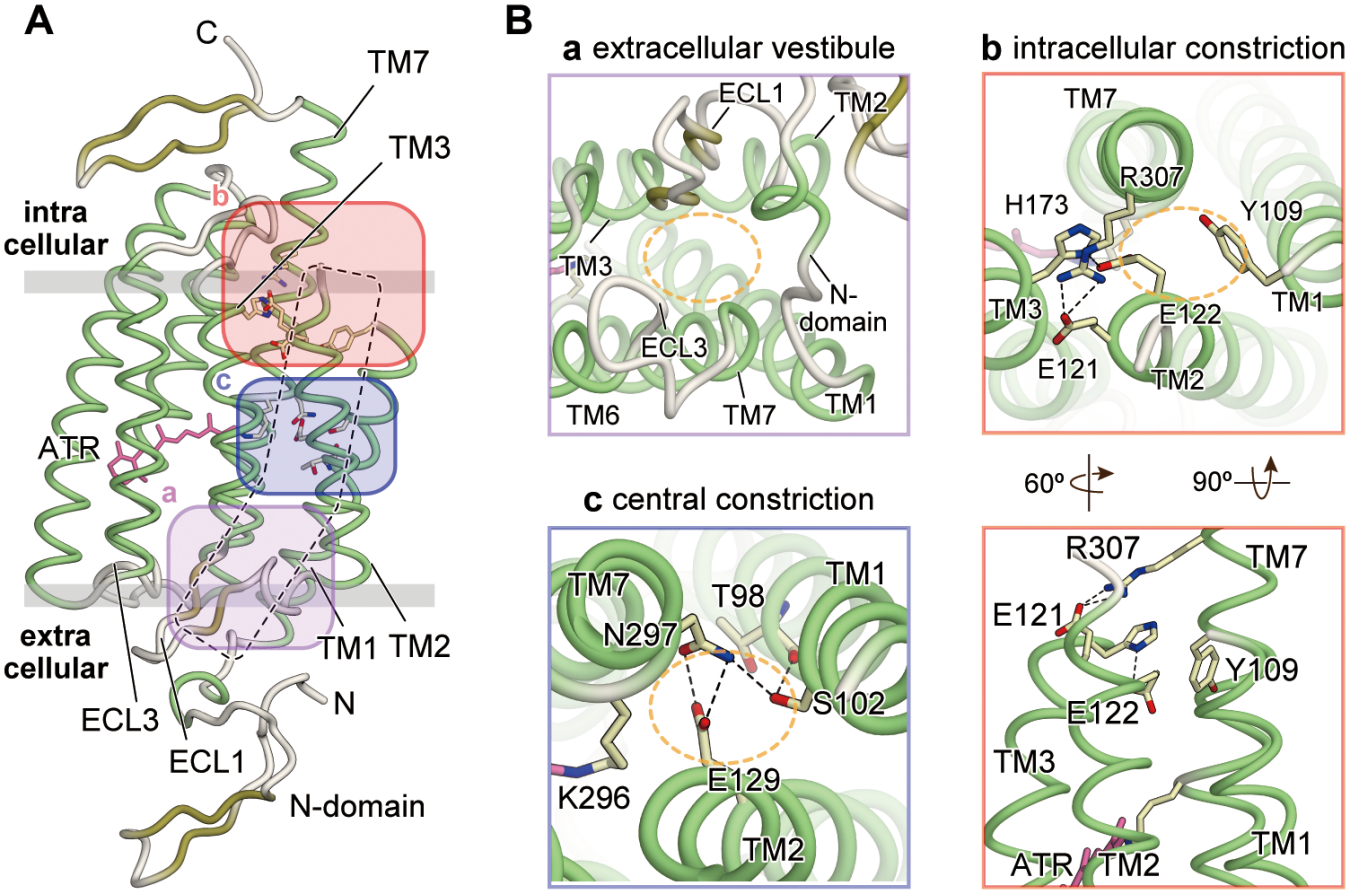
The two constrictions observed in the crystal structure of C1C2. The two constrictions in the crystal structure of C1C2 (PDB ID 3UG9) and the electrophysiological analysis of the constrictions. (A) Overall structure of C1C2, viewed parallel to the membrane with the three key regions highlighted (magenta, blue, and red). The dashed area represents the putative ion-conducting pathway. (B) Magnified views of the highlighted regions in (A). Black dashed lines are hydrogen bonds, and orange dashed circles represent the putative conducting pathway.

In addition to these electrophysiological and spectroscopic analyses, the 2.3 Å resolution structure of the chimeric ChR of *C*. *reinhardtii* ChR1 and ChR2 (C1C2) provided detailed insights into the architecture of ChR, including its cation-conducting pathway [6]. ChR forms a dimer in this crystal structure, and the electronegative pore in each monomer, formed by TMs 1, 2, 3, and 7, probably functions as the cation-conducting pathway, consistent with the previous computational and electrophysiological studies [6,13–19] (Fig 1A). The crystal structure revealed that an extracellular vestibule, formed by the N-domain and extracellular loops 1 and 3, expands to a diameter of about 8 Å, and suggested that this passage would allow water molecules to move from the extracellular side to the middle of the pathway [6] (Fig 1B, panel a). In contrast to the extracellular half, the intracellular half of the pathway is occluded at two constrictions, revealing a closed state. This is consistent with the fact that C1C2 was crystallized and harvested in the dark. These two constrictions, called the intracellular and central constrictions (corresponding to Fig 1A 1b and 1c respectively), contain titratable residues, including Glu122 and Glu129, which are highly conserved in the ChR family, but not in other rhodopsin family members. These residues link the TM helices by hydrogen bonds and a salt bridge to occlude the conducting pathway, and these interactions are considered to be disrupted in the conducting state. Although the crystal structure of C1C2 identified certain residues that may be important for channel opening, it remains unknown how the residues in these constrictions prevent ion leakage in the ground state, and how the retinal isomerization induces the conformational change towards channel opening.

In this study, we investigated the functional roles of Glu122 and Glu129 in the two constrictions, by electrophysiological analyses and molecular dynamics (MD) simulations. The results revealed that Glu129 in the central constriction is important for preventing ion leakage in the ground state. In addition, we analyzed the conformational change upon retinal isomerization, which is the very first stage in the photocycle, by performing the MD simulation with 13-*cis* retinal-bound C1C2.

## Materials and Methods

### Initial structures and system preparation

The atomic coordinates of the crystal structure of ChR (Protein Data Bank (PDB) entry 3UG9 [6]) were retrieved from the PDB. Two disordered regions (Gly110-Thr117 and Glu332-Asn327) were modeled with the program Modeller [20]. The protonation state of each glutamate or aspartate was determined based on the pattern of hydrogen bonds observed in the crystal structure, as well as the results of the PROPKA calculation [21], except for Glu163, which was reported to be deprotonated in the ground state [22]. The phosphoryloleoyl phosphatidylcholines (POPCs) were generated by using the VMD plugin [23]. The full simulation system included ChR, POPC, water molecules, and chloride and sodium ions. The system size was 128 (Å) × 128 (Å) × 128 (Å), and contained about 190,000 atoms.

All of the water molecules observed in the crystal structure were kept. The missing hydrogen atoms were built with the program VMD [23]. An explicit solvent periodic boundary system was prepared. The net charge of the simulation systems was neutralized through the addition of chloride and sodium ions. The molecular topologies and parameters from the Charmm36 force field [24] were used for the protein, lipid, and water molecules, and the previously reported values [25–28] were used for the retinal molecules.

### Molecular dynamics simulations

Molecular dynamics simulations were performed with the program NAMD 2.8 [29]. The systems were first energy minimized for 1,000 steps with fixed positions of the non-hydrogen atoms, and then for another 1,000 steps with 10 kcal/mol restraints for the non-hydrogen atoms, except for the lipid molecules within 5.0 Å from the proteins. Next, equilibrations were performed for 0.01 ns under NVT conditions, with 10 kcal/mol restraints for the heavy atoms of the protein. Finally, equilibrations were performed for 0.5 ns under NPT conditions with the 1.0 kcal/mol restraints. In the equilibration and production processes, the pressure and temperature were set to 1.0 atm and 300 K, respectively. Constant temperature was maintained by using Langevin dynamics. Constant pressure was maintained by using the Langevin piston Nose-Hoover method [30]. Long-range electrostatic interactions were calculated by using the particle mesh Ewald method [31]. We performed each simulation at least twice with different initial velocities, and obtained similar results from these trajectories.

### Modeling of 13-*cis* retinal bound C1C2

The coordinates of Lys-13-*cis*R were obtained from the K-intermediate of bacteriorhodopsin (PDBID: 1IXF [32]). The main chain atoms (i.e., the C, O, N, and CA atoms) and the β-ionone ring of this Lys-13-*cis*R molecule were superimposed on those of Lys296-ATR of C1C2, and then the Lys296-ATR of C1C2 was replaced with the Lys-13-*cis*R. After this replacement, 1,000 steps of energy minimization were performed with fixed positions of the non-hydrogen atoms. Subsequently, another 1,000 steps of energy minimization were performed with 10 kcal/mol restraints for the non-hydrogen atoms, except for the lipid molecules within 5.0 Å from the protein. The equilibration and production runs were performed in the same manner as described above. The topology and force field parameters for 13-*cis* retinal were the same as those for ATR, which are also compatible with 13-*cis* retinal.

### Trajectory analysis

The residue-averaged root mean square deviation (RMSD) and the correlation matrix for all Cα atoms were calculated as described previously [33]. The RMSD calculations were performed after superimposing each trajectory frame on transmembrane helices 1–7 of the crystal structure. The correlation coefficient calculations were performed after superimposing each trajectory frame on transmembrane helices 3–5 of the crystal structure.

### Electrophysiology

For electrophysiology, HEK293 cells were cultured on poly-lysine-coated, glass bottom culture dishes (Matsunami), and were transfected with 0.1 mg of a plasmid construct encoding the GFP-tagged C1C2 or the GFP-tagged C1C2 mutants. At 24–30 h after transfection, the cells were placed in bath medium, containing 140 mM NaCl, 1 mM CaCl_2_, 2 mM MgCl_2_, 10 mM HEPES, and 5 mM glucose (pH 7.4 with NaOH), under an inverted microscope (Olympus IX71). A borosilicate patch pipette (Harvard Apparatus), with a resistance of about 5–8 MΩ, was filled with 140 mM KCl, 5 mM EGTA, 2 mM MgCl_2_, and 10 mM HEPES (pH 7.2 with KOH). The C1C2 currents were recorded in both the voltage-clamp mode and whole-cell configuration. The cells were held at a membrane potential of -80 mV, and were depolarized by 10 mV voltage steps of 1.8 s up to 170 mV. The light-dependent currents were activated 200 ms after the depolarization step, with 465 nm light (1.5 mWmm^-2^) for 1,000 ms, elicited by a high power LED illumination system (LEX2-B, Brainvision) connected to an A/D converter (Digidata 1440, Axon CNS, Molecular Devices), and controlled by the pClamp10 software (Axon CNS, Molecular Devices). Currents were measured using an Axopatch 200B amplifier (Axon CNS, Molecular Devices), filtered at 2 KHz and sampled at 5 KHz, using a Digidata 1440A digitizer (Axon CNS, Molecular Devices) controlled by the pClamp10 software (Axon CNS, Molecular Devices).

### Fluorescence measurements

To estimate the membrane expression levels of C1C2 and its mutants, the ratio of the membrane and cytosolic fluorescence values was determined. The cells were transfected with 0.1 mg WT C1C2 or C1C2 mutants, using Fugene 6, and cultured for 30 h. They were then washed with PBS, fixed with 4% paraformaldehyde in PBS for 20 min at room temperature (20°C), and washed again with PBS before microscopy observations. GFP fluorescence was observed with a laser confocal microscope (FV1000 Olympus).

## Results and Discussion

### Deprotonation of Glu122 and Glu129 is important for channel activity

**Fig 2:**
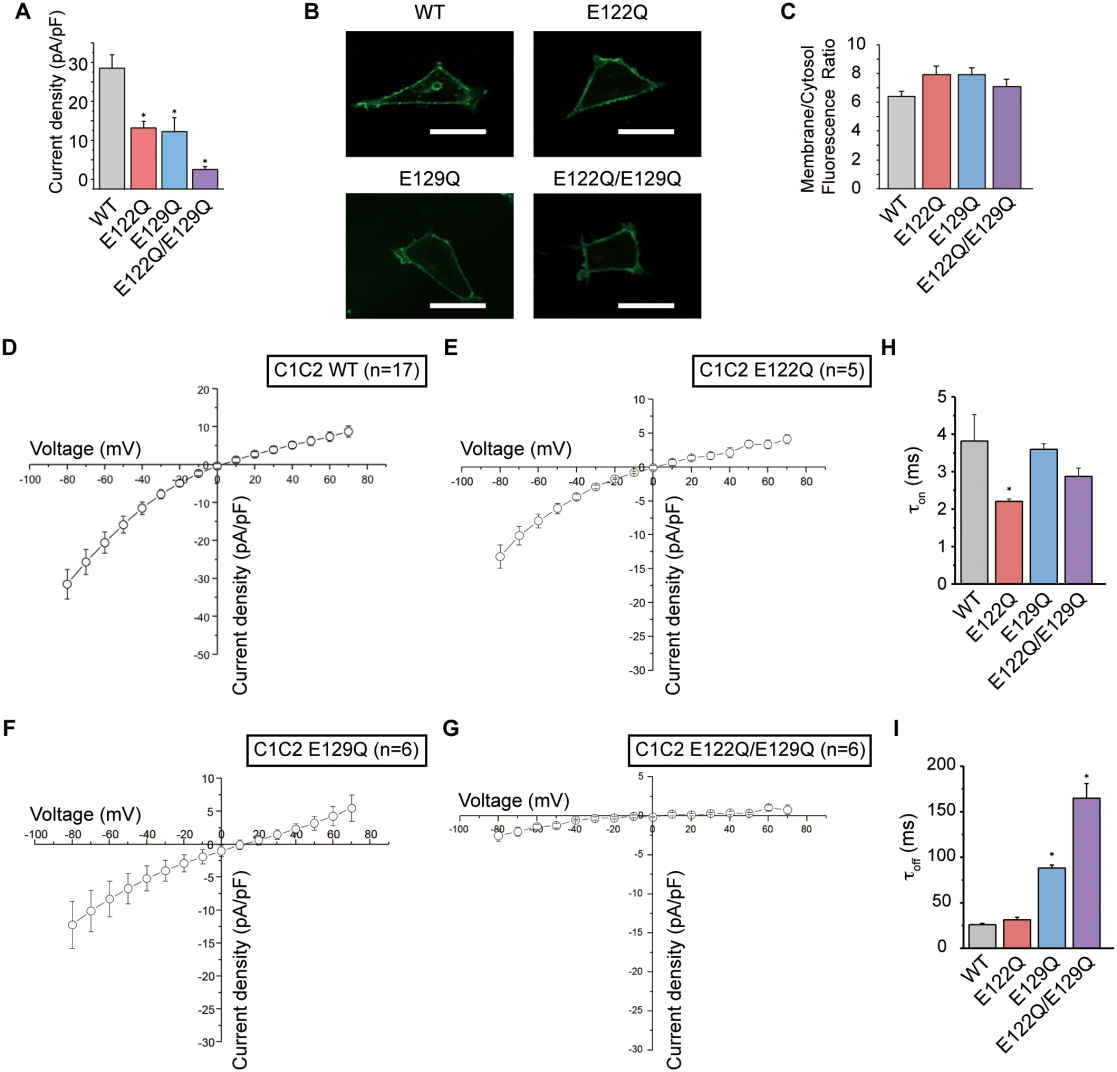
Electrophysiological analysis for C1C2 variants. (A) The peak amplitudes of the photocurrents, normalized by the cell’s input capacitance. (B) Conforcal images of representative HEK293 cells expressing the C1C2 WT and its mutants. Scale bar represents 30 μm. (C) The expression level of each C1C2 variant measured by the membrane/cytosol ratio of GFP fluorescence. (D-G) The current-voltage (*I-V*) relation curves for each mutant. (H, I) The kinetic parameters for each mutant, (H) opening rates (τ_on_) and (I) closing rates (τ_off_). The error bars represent s.e.m. of 3 experiments (n = 5–17 cells). * p < 0.05.

The crystal structure of the chimeric ChR of ChR1 and ChR2 (C1C2) revealed the central and intracellular constrictions in the pore [6]. In the intracellular constriction, the salt bridge between Glu121 and Arg307 links TM2 to TM7, and the hydrogen bond between Glu122 and His173 links TM2 to TM3 (Fig 1B panel b). In the central constriction, the hydrogen bond between Glu129 and Asn297 links TM2 to TM7 and occludes the pore (Fig 1B panel c). In this crystal structure, hydrogen bonds are formed between Glu122 and His173, and Glu129 and Asn297, suggesting the protonation of the side chains of Glu122 and Glu129 [6]. To clarify the functional importance of the protonation states of these glutamate residues, we prepared the E122Q and E129Q mutants, expressed them in HEK293 cells, and recorded their photocurrents in response to 465-nm light pulses. The results revealed that the E122Q and E129Q single mutants each showed a significant decrease in the photocurrent, and that the double mutation E122Q/E129Q almost completely abolished the photocurrent (Fig 2A and 2D–2G), despite their stable membrane expression (Fig 2B and 2C). Given that the glutamine mutation can mimic the protonated glutamic acid, these results suggested that Glu122 and Glu129 (Glu83 and Glu90 in ChR2) are deprotonated during the photocycle. These findings are consistent with previous spectroscopic studies showing that Glu90 in ChR2 is protonated in the dark/closed state, and its protonation state changes during the photocycle [13,34]. In addition, the calculation of the kinetic parameters, τ_on_ and τ_off_, showed that the E122Q and E129Q mutations affected the τ_on_ and τ_off_ values, respectively (Fig 2H and 2I).

### Deprotonation of both Glu122 and Glu129 results in proton leakage in the ground state

To investigate the effects of the protonation states of Glu122 and Glu129 on the ChR structure, we created atomic models of C1C2 with different protonation states based on the crystal structure (PDB entry 3UG9 [6]), and performed all-atom MD simulations in the presence of a 1-palmitoyl-2-oleoyl-sn-glycero-3-phosphocholine (POPC) lipid bilayer.

**Fig 3:**
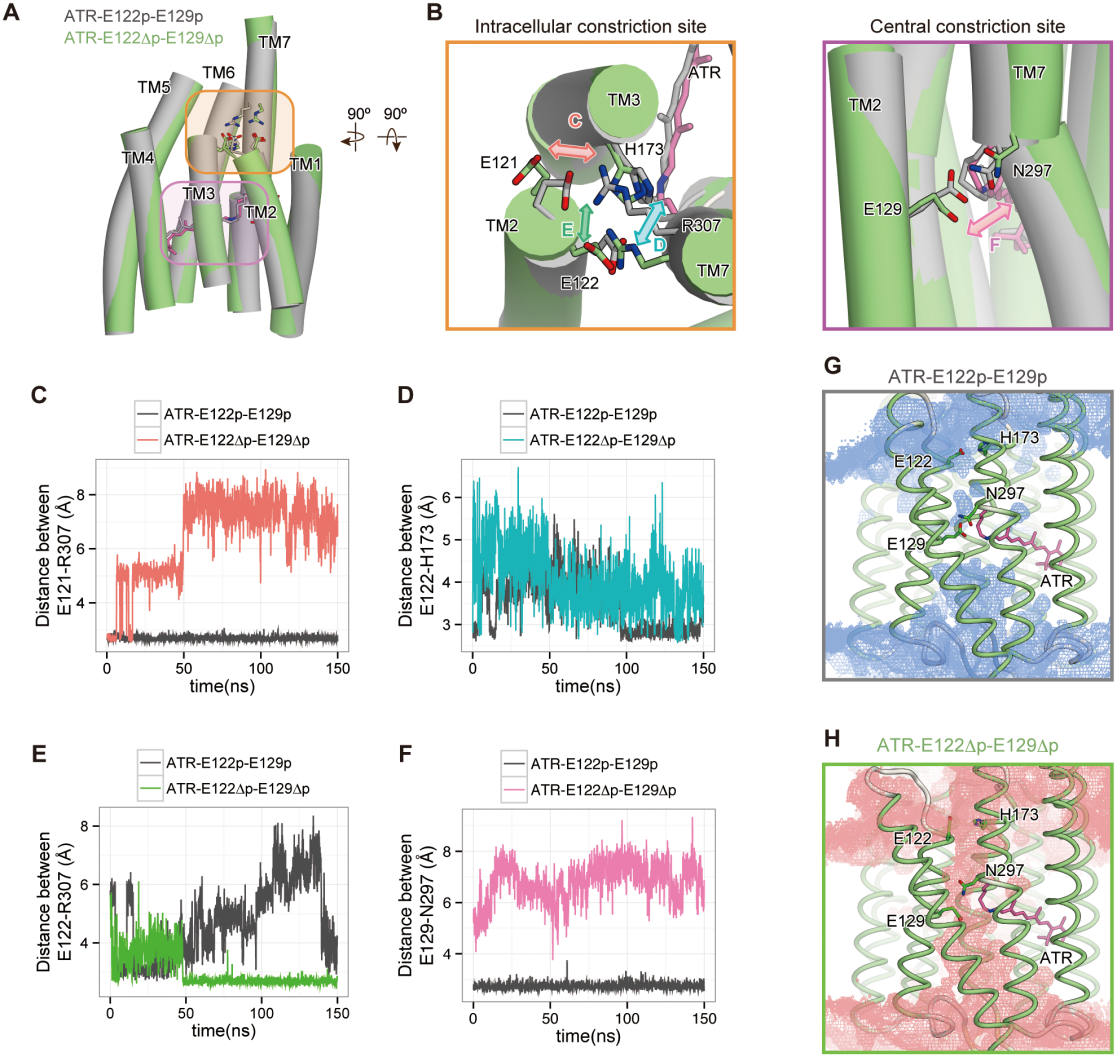
Effects of deprotonation of both Glu122 and Glu129 in the ground state. Structural comparison of the intracellular constrictions between the ATR-E122p-E129p and ATR-E122Δp-E129Δp simulations. (A) Overall structures of snapshots from the last frame of both simulations. Key residues are highlighted in orange and magenta. (B) Magnified view of the intracellular and central constrictions (left and right panels, respectively). Double arrows indicate the possible motions of Glu121-Arg307 (red), Glu122-His173 (cyan), Glu122-Arg307 (green) and Glu129-Asn297 (magenta). (C-F) Distances between (C) Glu121-Arg307, (D) Glu122-His173, (E) Glu122-Arg307, and (F) Glu129-Asn297. (G, H) Distribution of water molecules in the (G) ATR-E122p-E129p and (H) ATR-E122Δp-E129Δp simulations. The distribution maps are contoured at the probability density of 0.0015 molecules Å^-3^ ns^-1^. The time-averaged structure of the protein over 150 ns is shown.

We first performed the ATR-E122p-E129p simulation (Table 1). In this simulation, the overall structure was stable, and no large structural changes were observed (Fig 3A; gray). The interactions in both the central and intracellular constrictions were also preserved during the 150-ns simulation. The salt bridge between Glu121 and Arg307 in the intracellular constriction, and the hydrogen bond between Glu129 and Asn297 in the central constriction, were stable (Fig 3C and 3F; gray line). The hydrogen bond between Glu122 and His173 in the intracellular constriction was disrupted and reformed again during the 150-ns simulation (Fig 3D). As a result, a small number of water molecules entered through the intracellular constriction (Fig 3G). The extracellular side of the channel pathway, especially the extracellular vestibule observed in the crystal structure, was filled with water molecules (Fig 3G). However, no water channel was formed between the intra- and extracellular sides of the membrane, due to the stable hydrogen-bonds of the central constriction.

**Table 1:** Simulation systems used in this research.

**Simulation Name**	**retinal**	**E122**	**E129**	**time**
**ATR-E122p-E129p**	ATR	p	p	150 ns
**ATR-E122Δp-E129Δp**	ATR	Δp	Δp	150 ns
**ATR-E122p-E129Δp**	ATR	p	Δp	150 ns
**ATR-E122Δp-E129p**	ATR	Δp	p	150 ns
**13-** ***cis*** **R-E122Δp-E129p**	13-*cis*R	Δp	p	150 ns

The simulations performed in this research. In this table, “p” and “Δp” refer to protonated and deprotonated glutamate, respectively.

We next compared the results of the ATR-E122p-E129p and ATR-E122Δp-E129Δp simulations (Table 1), to examine the effects of the protonation states of Glu122 and Glu129. In the ATR-E122Δp-E129Δp simulation, the overall structure was stable (Fig 3A; green), but several changes in the intracellular and central constrictions were observed. In the intracellular constriction, the salt bridge between Glu121 and Arg307 was also stable over the 150-ns simulation (Fig 3C; red line), while the hydrogen bond between Glu122 and His173 was disrupted, as a result of the deprotonation of Glu122 (Fig 3D; cyan line). Instead, the deprotonated Glu122 formed a stable salt bridge with the side chain of Arg307 (Fig 3E; green line). In the central constriction, the hydrogen bond between Glu129 and Asn297 was disrupted upon the Glu129 deprotonation (Fig 3F; magenta line). The water distribution in the ATR-E122Δp-E129Δp simulation revealed that water molecules from both the intracellular and extracellular entrances filled the channel pathway, which resulted in the formation of the water-mediated hydrogen-bond network connecting the intra- and extracellular sides of the membrane (Fig 3H). The formation of this water-mediated hydrogen-bond network in the ground state may result in the leakage of protons across the membrane, without channel activation. Therefore, the protonation of Glu122 and/or Glu129 is important for preventing ion leakage in the ground state.

### Glu129 protonation is important for preventing proton leakage

**Fig 4:**
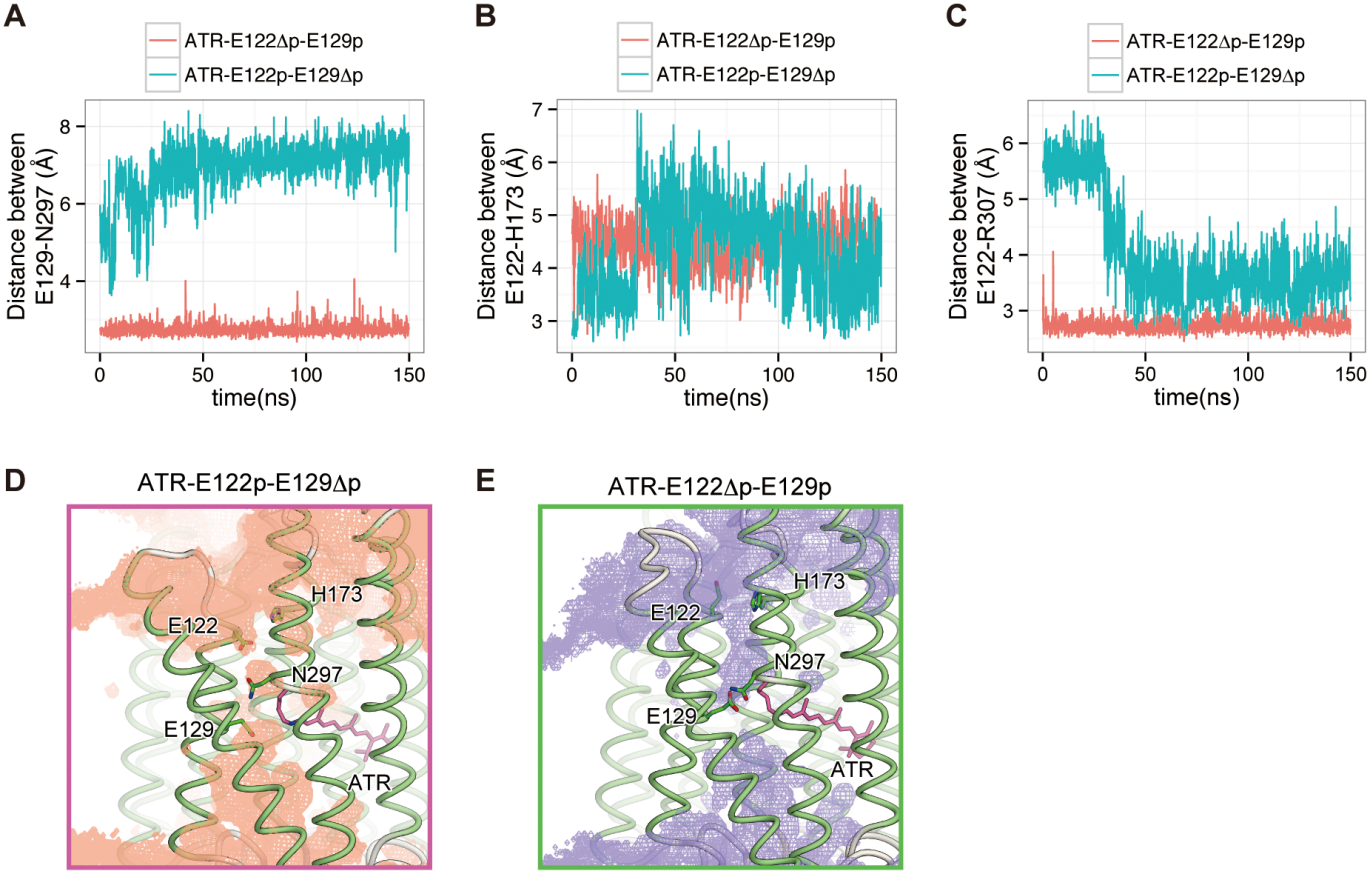
Effects of the deprotonation of either Glu122 or Glu129 in the ground state. (A, B, C) Distances between (A) Glu129-Asn297, (B) Glu122-His273 and (C) Glu122-Arg307 in the ATR-E122p-E129Δp and ATR-E122Δp-E129p simulations. (D, E) Distributions of water molecules in the ATR-E122p-E129Δp and ATR-E122Δp-E129p simulations. The distribution map is contoured at the probability density of 0.0015 molecules Å^-3^ ns^-1^. The time-averaged structure of the protein over 150 ns is shown.

To investigate the effect of the deprotonation of each glutamate residue, we performed simulations in which either Glu122 or Glu129 was deprotonated (ATR-E122Δp-E129p and ATR-E122p-E129Δp; Table 1). In these simulations, similar structural changes to those in the ATR-E122Δp-E129Δp simulation were observed: the Glu129-Asn297 hydrogen bond was disrupted upon Glu129 deprotonation in the ATR-E122p-E129Δp simulation (Fig 4A; cyan line), while the Glu122-His173 hydrogen bond was disrupted and the salt bridge between Glu122 and Arg307 was formed upon Glu122 deprotonation in the ATR-E122Δp-E129p simulation (Fig 4B and 4C; red line). However, the water distributions revealed that no water channels were formed in these simulations, suggesting that ion or proton leakage did not occur (Fig 4D and 4E). In the ATR-E122p-E129Δp simulation, the water permeation was disturbed by the interaction between Glu122 and His173, and a small number of water molecules entered through these constrictions, similar to the ATR-E122p-E129p simulation. In the ATR-E122Δp-E129p simulation, the hydrogen bond between Glu129 and Asn297 blocked the water permeation. In contrast to the case of the Glu122-His173 interaction, no water molecules passed through the channel pathway, because of the stable Glu129-Asn297 hydrogen bond. Therefore, we hypothesized that either Glu122 or Glu129 is protonated, to prevent the water channel formation and proton leakage.

A recent structural analysis of the C1C2 variant under neutral-pH conditions suggested that Glu122 is deprotonated in the ground state [35]. In this C1C2 variant, although the overall structure is essentially the same as that of the previous C1C2 structure obtained under low-pH conditions, the hydrogen bond between Glu122 and His173 is absent, and instead Glu122 forms a salt bridge with Arg307. This result suggested that Glu122 is deprotonated and interacts with Arg307, rather than His173, under neutral-pH conditions, as observed in the ATR-E122Δp-E129Δp and ATR-E122Δp-E129p simulations (Fig 3D, 3E and Fig 4B, 4C). Therefore, we concluded that Glu122 is deprotonated in the ground state, and that the protonated Glu129 and its hydrogen bond with Asn297 are important for preventing ion leakage across the membrane.

Our electrophysiological analysis revealed the decreased photocurrent in the E122Q mutant (Fig 2A). Given the structural similarity between glutamine and protonated glutamic acid, a transient interaction between Gln122 and His173 may occur in this mutant, as observed in the ATR-E122p-E129p and ATR-E122p-E129Δp simulations (Fig 3D and Fig 4B). In these Glu122 protonated simulations, the interaction between Glu122 and His173 restricted the entrance of water molecules through the intracellular constriction (Fig 3G; Fig 4D). Therefore, in the E122Q mutant, a similar interaction between Gln122 and His173 might disturb the water and ion permeation at the intracellular constriction (Fig 3G), resulting in the decreased photocurrent of the E122Q mutant.

Our electrophysiological analysis also revealed the decreased photocurrent and increased τ_off_ parameter of the E129Q mutant (Fig 2A and 2I), suggesting the importance of Glu129 deprotonation during the photocycle. Consistent with our proposal, recent studies showed that Glu129 is deprotonated and negatively charged in the conducting state, which is important for the ion selectivity [13,36–38]. However, the timing of the Glu129 deprotonation in the photocycle still remains controversial. Notably, some studies showed that Glu129 (Glu90) is deprotonated in the early stage of the photocycle of ChR2 [13,36], while others indicated that it is deprotonated in the desensitized stage of the photocycle in both ChR2 and C1C2 [37,38]. The results of our electrophysiological analysis of the E129Q mutant are consistent with the latter results, since the mutation affected τ_off_, rather than τ_on_ (Fig 2H and 2I). It should be noted here that a recent study suggested that the timing of the Glu129 (or Glu90 in ChR2) deprotonation is different between the ChR2 and ChR1/ChR2 chimeras, including C1C2 [38]. Further spectroscopic and crystallographic analyses of ChR may be required.

In addition, a recent study demonstrated that the replacement of Glu90 in ChR2 with lysine or arginine converted ChR2 into a chloride-selective channel [39]. Another report also revealed that the replacement of several residues in C1C2, including Glu129, converted it into an anion-selective channel [40]. In these anion-selective ChR variants, different hydrogen bond and/or salt bridge pairs from Glu129 and Asn297 might prevent ion leakage in the ground state.

### ATR isomerization to 13-cisR results in the movement of the cytoplasmic regions of TM6 and TM7

**Fig 5:**
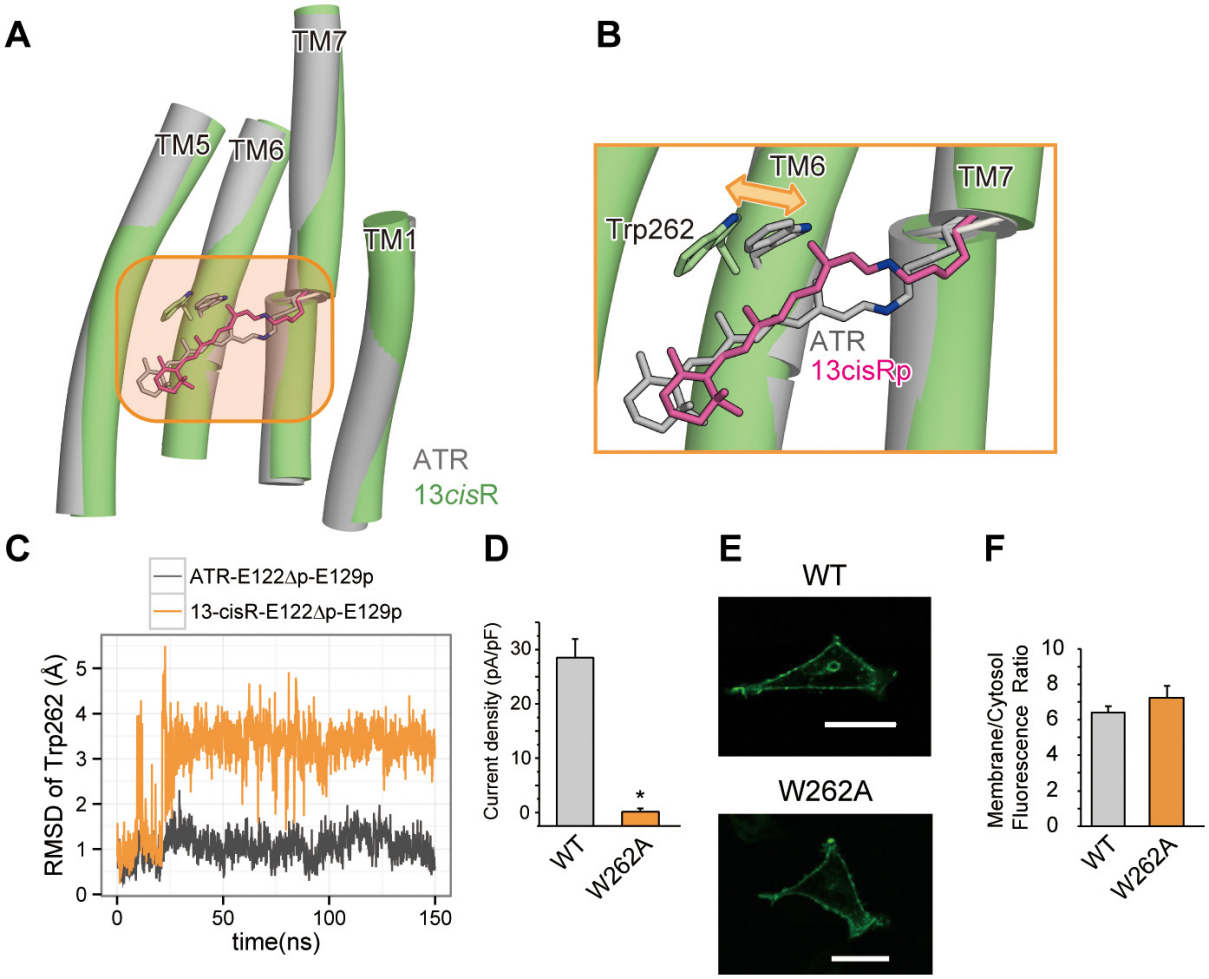
The conformational change in Trp262 upon retinal isomerization. (A) Structural comparison between the snapshots from the ATR-bound (grey) and 13-*cis*R-bound (green) simulations. (B) Magnified view of retinal and Trp262, from the orange-highlighted region in the left panel. Double arrows indicate the possible motions of Trp262. (C) The RMSD values of the Trp262 atoms, relative to those of the crystal structure. (D) The peak amplitudes of the photocurrents, normalized by the cell’s input capacitance. (E) Conforcal images of representative HEK293 cells expressing the C1C2 WT and W262A mutants. Scale bar represents 30 μm. (F) The expression level of W262A mutant measured by the membrane/cytosol ratio of GFP fluorescence. The error bars represent s.e.m. of 3 experiments (n = 5–17 cells). * *p* < 0.05.

**Fig 6:**
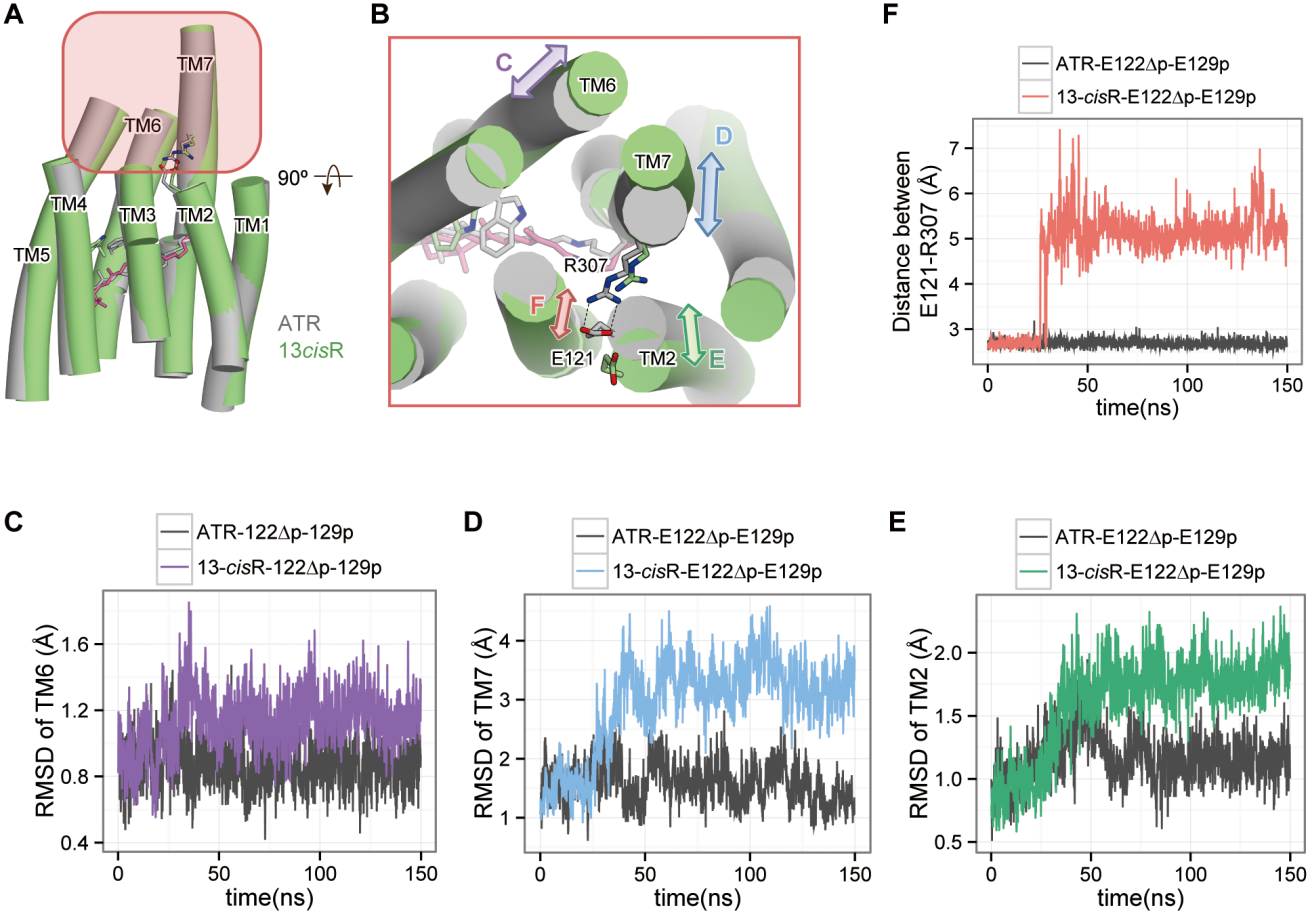
The movements of TM helices upon retinal isomerization. (A) Structural comparison between the snapshots from the ATR-bound (grey) and 13-*cis*R-bound (green) simulations. (B) Magnified cytoplasmic view of the red-highlighted region in the left panel. (C-E) The RMSD values of (C) TM6, (D) TM7 and (E) TM2, compared between the ATR-bound and 13-*cis*R-bound forms. (F) Distance between Glu121-Arg07 in the intracellular constriction.

The initial event of the photocycle, upon the absorption of blue light, is the isomerization of ATR to 13-*cis* retinal (13-*cis*R), which occurs on a femto-second time scale [8,10]. To investigate this initial conformational change in the photocycle, we performed the MD simulation of C1C2 with 13-*cis*R. The initial structure was modeled by simply replacing the ATR moiety of the crystal structure with 13-*cis*R, and subsequent energy minimization. We assumed that Glu122 was deprotonated in the ground state, considering the results of the ATR-E122Δp-E129p simulation, as described above. In this 13-*cis*R bound simulation (13-*cis*R-E122Δp-E129p; Table 1), conformational changes in the TM domains were observed. The 13-methyl group of 13-*cis*R shifted toward the cytoplasmic side and pushed out the indole ring of Trp262 (Trp223 in ChR2) on TM6 (Fig 5A–5C). The local steric conflict of the 13-methyl group with Trp262 caused the subsequent movement of the cytoplasmic half of TM6 (Fig 6A–6C). This movement was consistent with previous studies of other microbial rhodopsins, such as bacteriorhodopsin (BR) and sensory rhodopsin II (SRII), which indicated that the retinal isomerization and the steric collision between the 13-methyl group of 13-*cis*R and the tryptophan residue on TM6 (Trp182 in BR, Trp171 in SRII and Trp262 in C1C2) cause the movements of TM6 and TM7 [25–28]. To verify the functional importance of Trp262, we measured the photocurrents of the W262A mutant of C1C2 in HEK293 cells, and found that this mutant completely abolished the photocurrent, despite its robust membrane expression (Fig 5D, 5E and 5F). These results suggested that the presence of a bulky side chain adjacent to the 13-methyl group of 13-*cis*R is important for triggering the channel opening.

**Fig 7:**
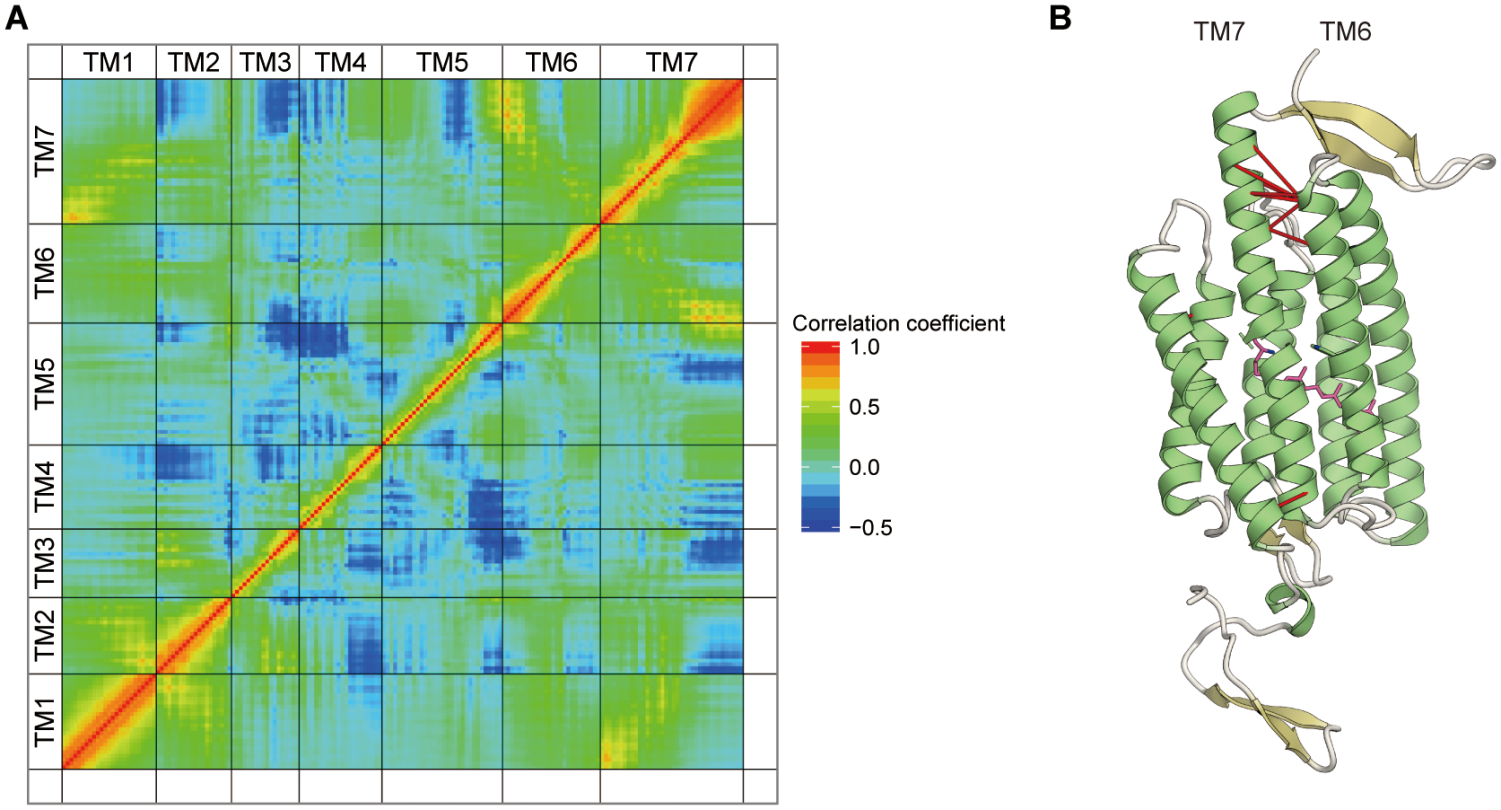
Correlation analysis for the 13-*cis*R-122Δp-129p simulation. (A) The matrix of correlation coefficients for the pairs of Cα atoms. (B) Mapping of the correlation coefficients to the structure. The black dashed circle represents the pair of Cα atoms with a correlation coefficient greater than 0.7. The red dashed circle represents the pair of Cα atoms in TM2 and TM7 that has a negative correlation coefficient.

In addition to the movement of TM6, the outward movement of the cytoplasmic half of TM7 was observed in the 13-*cis*R-E122Δp-E129p simulation (Fig 6B and 6D). To visualize the correlated motions of TM6 and TM7, we calculated the correlation coefficient between each Cα atom in the TM domains. The correlation matrix showed that the cytoplasmic halves of TM6 and TM7 have a strong correlation, with a coefficient greater than 0.7 (Fig 7; black dashed circle in panel A). Moreover, in this correlation matrix, we found that the cytoplasmic half of TM2 has a negative correlation coefficient with TM7 (Fig 7A; red dashed circle), indicating that the cytoplasmic halves of TM2 and TM7 move in opposite directions (Fig 6B and 6E). These movements of TM2 and TM7 facilitate the disruption of the salt bridge between Glu121 and Arg307 in the intracellular constriction (Fig 6F). Taken together, the results of the 13-*cis*R-E122Δp-E129p simulation suggested that retinal isomerization induces the series of conformational changes of Trp262, followed by those of TM6, TM7, and TM2, toward channel opening. This is consistent with previous results obtained by cryo-electron microscopy and DEER spectroscopy, which suggested the movements of TM2, 6 and 7 in ChR2 during the photocycle, based on projection difference maps [41–43].

Our 13-*cis*R-122Δp-129p simulation results suggested that the isomerization of retinal induces the conformational change of Trp262, which is followed by the subsequent movements of TM6, TM7 and TM2 (Figs 5 and 6). Given that the P_1_^500^ state in the photocycle is observed ~50 ns after the retinal isomerization [8,10] and that large conformational changes of the protein backbone were observed in this P_1_^500^ state by FTIR spectroscopy [8,44], the structural changes, including the movements of TM6, TM7 and TM2, observed in the 13-*cis*R-122Δp-129p simulation are likely to represent the structural transition from the ground state to the P_1_^500^ state. Moreover, this P_1_^500^ state is considered to be a non-conducting state [7–12], which is consistent with the observations in this simulation: the channel pore radii on both the cytoplasmic and extracellular sides were still comparable to those of the ATR-122Δp-129p simulation, and thus too narrow to permeate monovalent or divalent cations.

The events that occur after retinal isomerization (i.e., P_1_^500^) involve the proton transfer between the Schiff base of the retinal and the surrounding protein side chains, which finally results in the ion-conducting state. An investigation of the subsequent events is beyond the scope of the present study, since the time scales on which those events occur are several tens of micro-seconds to milli-seconds [8,10,44], and thus exceed the current limitations of our computational resources. Nevertheless, the present study provides mechanistic insights into the nature of the two constrictions and the early response of the protein conformations to the isomerization of the retinal chromophore, and will serve as a framework for future investigations of the formation of the ion-conducting state.
